# Combination Lipid-Lowering Therapy After ACS: Should this be the New Standard of Care?

**DOI:** 10.1007/s11886-026-02346-8

**Published:** 2026-02-03

**Authors:** Julia Brandts, Nikolaus Marx

**Affiliations:** 1 Department of Internal Medicine I, Cardiology, RWTH Aachen University, Pauwelsstraße 30, Aachen, 52074 Germany; 2 Department of Primary Care and Public Health, School of Public Health, Imperial College London, London, UK

**Keywords:** ACS, Lipid-lowering therapy, LDL-cholesterol targets, Statins

## Abstract

**Purpose of Review:**

To examine whether early combination lipid-lowering therapy after acute coronary syndrome (ACS) should be considered as a standard approach. The review assesses current international guideline recommendations, supporting clinical evidence, and real-world implementation gaps.

**Recent Findings:**

Recent European and U.S. guidelines emphasise lower low-density lipoprotein-cholesterol (LDL-C) targets and suggest earlier use of combination therapy. Data from randomised trials, registry-based emulations, and observational cohorts demonstrate that early initiation of high-intensity regimens improves LDL-C goal attainment and may reduce the recurrence of cardiovascular events. However, registry data reveal substantial global undertreatment and limited treatment intensification.

**Summary:**

Early combination therapy represents a pragmatic strategy to accelerate LDL-C goal achievement after ACS. While this approach could narrow current treatment gaps, it must be balanced against the trend toward personalised, risk-based lipid management. Future research should address how simplified treatment algorithms can coexist with individualised care frameworks.

## Introduction

Patients with recent acute coronary syndrome (ACS) face rates of cardiovascular death, myocardial infarction, and ischemic stroke approaching ~ 10–18% in the year after hospitalisation [1–3], underscoring the need for rapid and profound control of the underlying disease, which is atherosclerosis. Therefore, optimisation of low-density lipoprotein-cholesterol (LDL-C), as a cumulative and causal factor in atherosclerosis development, remains central to secondary prevention. Meta-analyses across randomised controlled trials consistently demonstrate that cardiovascular benefit correlates with the absolute magnitude of LDL-C reduction achieved, with similar relative risk reductions per unit LDL-C lowering observed regardless of the underlying therapeutic mechanism [4–6].

This log-linear relationship has informed increasingly stringent guideline targets. ESC recommendations since 2019 specify an LDL-C level of < 1.4 mmol/L (55 mg/dL) with a > 50% reduction from baseline, and < 1.0 mmol/L (40 mg/dL) in those with recurrent events [7–10]. The 2025 ACC/AHA ACS guidelines similarly strengthen this approach, recommending high-intensity statin therapy for all ACS patients, with the option to initiate concurrent ezetimibe, and emphasising that non-statin agents are indicated when LDL-C remains ≥ 1.8 mmol/L (70 mg/dL) despite maximally tolerated statin therapy [11]. In parallel, the 2025 ESC/EAS Focused Update introduces explicit ACS recommendations with the consideration to intensify lipid-lowering therapy during the index hospitalisation and to consider initiating combination therapy with high-intensity statin plus ezetimibe in treatment-naïve patients who are unlikely to reach LDL-C goals on statin alone (Class IIa B recommendation) [7]. However, real-world implementation of intensive lipid management has lagged behind guideline recommendations, raising the question of whether a default combination therapy started in the hospital for patients after ACS is justified. In this review, we compare current guideline recommendations, compile the evidence on which these recommendations are based, and reflect on current real-world data and the implications that a standard combination therapy could have.

## Current Guideline Recommendations

Contemporary guidance converges on early initiation of high-intensity statin therapy after ACS, addition of non-statin agents based on on-treatment LDL-C and prior therapy, and scheduled lipid reassessment (Table 1). The ESC 2023 ACS guideline recommends starting a high-intensity statin as early as possible (ideally pre-PCI), re-checking lipids 4–6 weeks after each change, considering in-hospital ezetimibe when targets are unlikely to be achieved with statin alone, and initiating a PCSK9 inhibitor during hospitalization for patients already on statin plus ezetimibe who remain above goal; subsequent 4–6-week checks guide further adjustments [10]. The ACC/AHA 2025 ACS guideline similarly recommends high-intensity statin for all and adding a nonstatin when LDL-C ≥ 70 mg/dL (≥ 1.8 mmol/L) on maximally tolerated statin, with further intensification reasonable at 55–69 mg/dL in very-high-risk patients; concurrent statin + ezetimibe may be considered, and lipids are reassessed at 4–8 weeks to direct intensification (ezetimibe, PCSK9 mAb, inclisiran, or bempedoic acid as appropriate) [11]. The ESC/EAS 2025 Focused Update (Dyslipidaemia) adds ACS-specific recommendations that upfront combination therapy (high-intensity statin plus ezetimibe) should be considered in treatment-naïve patients when the anticipated LDL-C reduction with statin alone is insufficient to achieve goals, and that intensification during the index hospitalisation is recommended for patients who were on any LLT with priorisation of agents with cardiovascular outcome benefit (ezetimibe, PCSK9 monoclonal antibodies, and bempedoic acid).

**Table 1:** Initial in-hospital therapy

Treatment at Admission	2025 ACC/AHA ACS Guideline	2023 ESC ACS Guideline	2025 ESC/EAS Focused Update (Dyslipidaemia)
Naïve or on lOW-/MODERATE INTENSITY STATIN	• High-intensity statin for all ACS patients (atorvastatin 40–80 mg or rosuvastatin 20–40 mg). • Concurrent ezetimibe (Class 2b).	• High-intensity statin started as early as possible, ideally before PCI (Class I C). • Concurrent ezetimibe (Class IIa B).	• High-intensity statin started as early as possible (Class I C). • If not expected to achieve the LDL-C goal with statin therapy alone: Concurrent ezetimibe (Class IIa B).
On maximally tolerated Statin	• If LDL-C < 55 mg/dL (< 1.4 mmol/L): Continue therapy (Class 1) • If LDL-C ≥ 70 mg/dL (≥ 1.8 mmol/L): add non-statin (ezetimibe, PCSK9 mAb, inclisiran, or bempedoic acid) (Class 1) • If LDL-C 55–69 mg/dL (1.4–<1.8 mmol/L) → addition of non-statin (ezetimibe, PCSK9 mAb, inclisiran, or bempedoic acid) is reasonable (Class 2a).	• If LDL-C < 55 mg/dL (< 1.4 mmol/L): Continue therapy • If LDL-C ≥ 55 mg/dL (≥ 1.4 mmol/L): add ezetimibe (Class I)	• Intensification of lipid-lowering therapy to further lower LDL-C levels (Class I, C) by adding non-statin therapies with proven cardiovascular benefit (ezetimibe, a PCSK9 mAb, or bempedoic acid)
On maximally tolerated Statin and ezetimibe	No explicit statement	• If LDL-C < 55 mg/dL (< 1.4 mmol/L): Continue therapy • If LDL-C ≥ 55 mg/dL (≥ 1.4 mmol/L): add PCSK9i (Class I)	• Intensification of lipid-lowering therapy to further lower LDL-C levels (Class I, C) by adding non-statin therapies with proven cardiovascular benefit (a PCSK9 mAb, or bempedoic acid) the choice should be based on the magnitude of additional LDL-C lowering needed
Statin intolerant	Non-statin therapy (ezetimibe, PCSK9, inclisiran, or bempedoic acid) recommended to lower LDL-C (Class 1).	No explicit statement for ACS patients	No explicit statement for ACS patients • non-statin therapies alone or in combination with proven cardiovascular benefit (ezetimibe, a PCSK9 mAb, or bempedoic acid) (Class I, A), the choice should be based on the magnitude of additional LDL-C lowering needed

**key: **ACS, Acute Coronary Syndrome; LDL-C, Low-Density Lipoprotein Cholesterol; PCI, Percutaneous Coronary Intervention; PCSK9, Proprotein Convertase Subtilisin/Kexin Type 9; PCSK9i, PCSK9 Inhibitor; mAb, Monoclonal Antibody

Therefore, contemporary guidelines endorse fast, deep LDL-C lowering post-ACS and suggest early combination therapy in selected patients.

## Evidence Base for Combination Therapy

### Combination of Lipid-lowering therapies after ACS and reduction of CV Events

Randomised evidence supports starting combination LLT in the early post-ACS window. In IMPROVE-IT, ezetimibe added within 10 days after ACS to background statin produced a modest but significant reduction in major adverse cardiovascular events (MACE) over six years, with similar relative benefit at lower baseline LDL-C strata. Complementary randomised data in chronic ASCVD can be derived from the RACING and LODESTAR trials [12, 13]. In RACING (moderate-intensity statin + ezetimibe vs. high-intensity statin), combination therapy achieved noninferior 3-year cardiovascular outcomes with comparable (often lower) achieved LDL-C and fewer treatment discontinuations/intolerance events [12]. In LODESTAR (treat-to-target strategy, typically using moderate-intensity statin ± ezetimibe to reach 55–70 mg/dL, vs. fixed high-intensity statin), the treat-to-target approach was noninferior for 3-year cardiovascular outcomes with similar mean on-treatment LDL-C and lower rates of new-onset diabetes, supporting the clinical utility of combination therapy to reach targets with less statin exposure when appropriate [13].

The CVO-Trial of alirocumab, Odyssey-Outcomes, showed additional event reduction when added in the months after ACS among patients with an LDL-C level of 1.8 mmol/l or higher despite maximally tolerated statin therapy, with larger absolute benefit the closer patients are enrolled to the index event [14]. Evolocumab in the FOURIER trial showed cardiovascular benefit in patients with atherosclerotic cardiovascular disease and LDL-C levels of 1.8 mmol/l or higher who were receiving statin therapy [15].

Where access or acceptance of injectables is limited, adding bempedoic acid to statin/ezetimibe is a practical route for further LDL-C reduction. CLEAR Outcomes showed a 13% MACE reduction in statin-intolerant patients (post-ACS within 90 days excluded), positioning bempedoic acid as an option for partial-intolerance or as a third oral agent while assessing eligibility for PCSK9-pathway therapy [16].

Together, these data support the use of a combination of lipid-lowering strategies in patients at risk of a recurrent ASCVD event, particularly those who have recently experienced an ACS.

### Lipid-lowering after ACS and Plaque Stabilisation

Mechanistic and imaging data provide biological plausibility for this clinical benefit. Intravascular imaging trials demonstrate faster plaque stabilisation with combination therapy in the early post-MI phase. In PACMAN-AMI, patients with recent AMI were randomised within 24 h of primary PCI to alirocumab or placebo on top of high-intensity rosuvastatin [17], which led to a between-group difference in achieved LDL-C of approximately − 51 mg/dL (−1.3 mmol/L) in favour of alirocumab. Over 52 weeks, percentage atheroma volume decreased by −2.13% vs. −0.92% (between-group difference − 1.21%, *P* < 0.001), and minimum fibrous-cap thickness, a marker of plaque stability, increased by + 62.7 μm vs. + 33.2 μm (difference + 29.7 μm, *P* = 0.001).

In HUYGENS, evolocumab was initiated within 10 days after NSTEMI on top of high-intensity statin therapy. After 50 weeks, plaque burden regressed by − 0.9% vs. − 0.3% (difference − 0.6%, *P* < 0.05) and fibrous-cap thickness increased by + 42.7 μm vs. + 18.4 μm (*P* < 0.001) [18].

Overall, these studies underscore that intensive lipid-lowering, started during or soon after the index ACS, achieves deeper LDL-C reductions and accelerates plaque stabilisation.

## Treatment Gaps in Real-World Practice

At the population level, mean untreated LDL-C concentrations of 3.0–3.5 mmol/L [19, 20] and target thresholds < 1.4 mmol/L mean that high-intensity statin therapy alone, typically providing ~ 50% reductions, will not achieve goal levels in most patients, making combination therapy necessary. European registries echo these findings: The Davinci Study (2017–2018), which included patients on LLT, showed that only 39% of patients with ASCVD would attain the 2016 ESC/EAS goal of < 1.8 mmol/L for ASCVD patients and 18% of patients the current goal of < 1.4 mmol/L, respectively [21]. The SANTORINI study (2020–2021), including patients at high and very high cardiovascular risk, also demonstrates suboptimal LDL-C goal attainment rates (20.7%) among patients with ASCVD. While combination therapy was associated with better LDL-C management, its adoption lagged, and it was used in 25.6% of patients [22]. Moreover, the one-year follow-up revealed that guideline-directed intensification remained infrequent with ~ 30%, despite LDL-C above targets [23]. Simulation studies suggest that through triple oral combination (high-intensity statin, ezetimibe and bempedoic acid) ~ 70% of patients could attain treatment target values [24]. The simulated addition of a PCSK9i to high-intensity statins and Ezetimibe could bring ~ 90% of patients to goal [25].

In the most recent international INTERASPIRE study spanning 13 countries across six WHO regions, less than one in five patients with a recent coronary event (elective CABG/PCI, STEMI/NSTEMI, or unstable angina) achieved the LDL-C goal of < 1.4 mmol/L; use of combination therapy and treatment intensification were low, with pronounced disparities by national income level [26]. Attendance at cardiac rehabilitation was also poor (≈ 9%), further limiting the “4–8 week” reassessment touchpoint assumed by many algorithms. Feeding into the observation of a failing stepwise approach, 66.3% of patients in INTERASPIRE, despite not achieving their goals, experienced no change in treatment intensity within the first 6–12 months after the index event.

Default step-up treatment algorithms, which depend on sequential reassessment and gradual intensification, may contribute to the observed delays in achieving LDL-C targets and at least partly explain the persistent treatment gap.

## Clinical Evidence and Practical Considerations for Upfront Combination Therapy

**Fig. 1:**
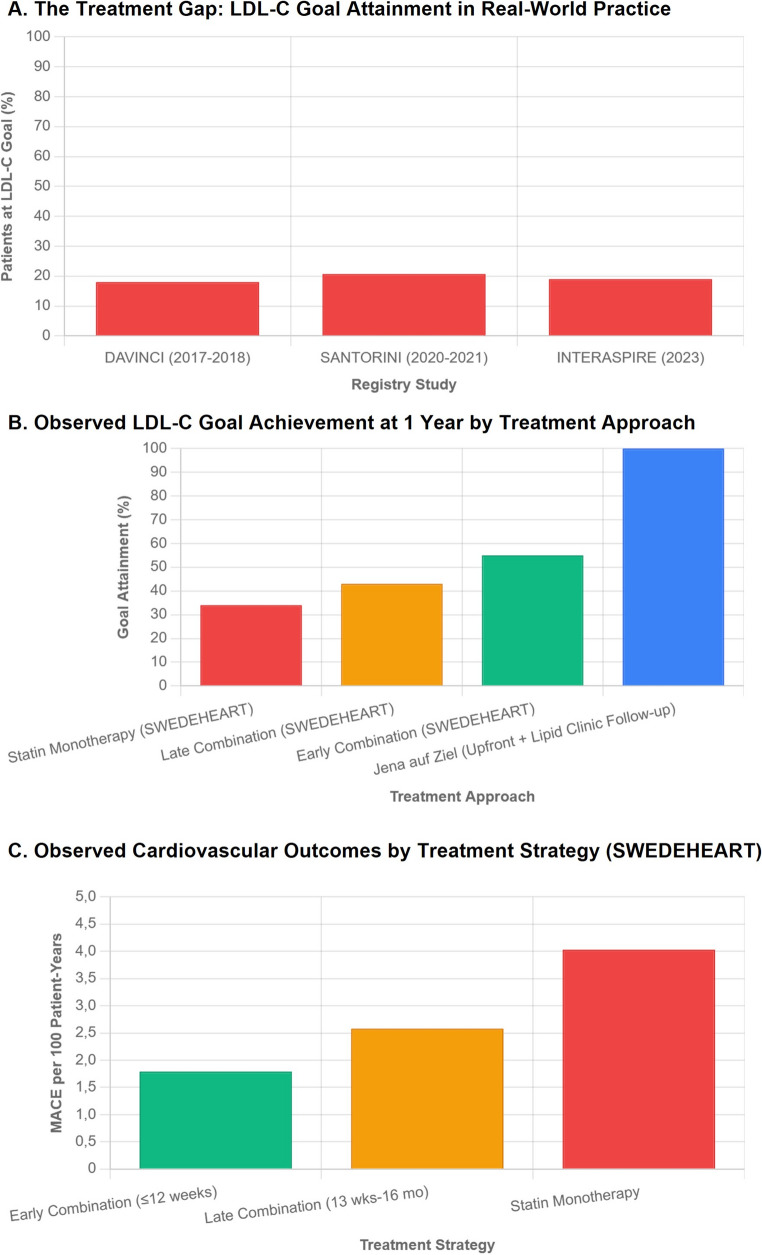
Early Combination Lipid-Lowering Therapy After Acute Coronary Syndrome: From Evidence to Implementation. Definitions used for early and late combination refer to the SWEDEHEART analysis by Leosdottir [29]: early combination = statin + ezetimibe initiated ≤ 12 weeks post-discharge) and late combination = statin + ezetimibe initiated 13 weeks up to 16 months post-discharge). LDL-C: low-density lipoprotein cholesterol

Guidelines currently recommend combination therapy in ACS patients whose baseline LDL-C is ≥ 1.8 mmol/L (70 mg/dL), or when treatment goals are unlikely to be reached with high-intensity statin alone. These recommendations exemplify the broader shift toward precision/individualised lipid management, where treatment selection is guided by patients’ baseline LDL-C and the predicted likelihood of goal attainment. This threshold-based approach avoids exposing patients with lower starting LDL-C levels to additional therapy; however, it also adds complexity to the care pathway, as treatment decisions must be tailored to individual baseline values and the expected magnitude of statin response. An alternative strategy would be to initiate oral dual combination therapy in all ACS patients, regardless of their baseline LDL-C levels, thereby simplifying the pathway and ensuring that more patients achieve the recommended LDL-C targets (Fig. 1).

Data supporting this strategy comes from regional initiatives such as the *Jena auf Ziel—JaZ* program, which enrolled 85 STEMI patients in 2021. All patients were started immediately on atorvastatin 80 mg plus ezetimibe 10 mg at admission, combined with structured education on cardiovascular risk modification and LDL-C target setting. LDL-C was measured at admission, during hospitalisation, and at discharge, and values were recorded on patient cards to facilitate follow-up. In those who did not reach the ESC/EAS target of < 1.4 mmol/L (< 55 mg/dL), therapy was escalated with bempedoic acid or PCSK9 inhibitors. This systematic approach resulted in 100% treatment goal attainment at 1 year and 60% at 2 years after ACS [27, 28]. The reduction in LDL-C goal attainment observed over the second year of follow-up was largely attributed to modifications in therapy after transitioning from specialist to primary care management. After 12 months, 38% of patients continued follow-up with their general practitioners, among whom 27.5% remained at target, compared with 72.5% of those followed in the dedicated lipid clinic. Mean LDL-C levels were correspondingly higher in the primary care group (2.1 ± 1.0 mmol/L) than in the lipid clinic group (1.2 ± 0.7 mmol/L; *p* < 0.01). The main determinant of non-goal attainment was de-intensification or discontinuation of LLT in community practice, whereas true patient non-adherence played a comparatively minor role. Contributing factors included differences between national and European guideline targets, reimbursement limitations for non-generic agents such as PCSK9 inhibitors and bempedoic acid, and less structured follow-up pathways in routine care. Nevertheless, despite this partial de-escalation of therapy after the first year, the proportion of patients maintaining LDL-C targets at two years (60%) in Jena auf Ziel remained substantially higher than the goal-attainment rates reported in contemporary European and international registries, where fewer than one in five patients typically reach the recommended LDL-C levels. This suggests that early combination therapy, coupled with structured follow-up, can achieve and sustain superior lipid control compared to current routine practice.

Data from the SWEDEHEART registry further underscore the prognostic relevance of early combination therapy in achieving lipid goals and improving cardiovascular outcomes [29]. In this analysis, 35,826 statin-naïve patients discharged on statin therapy after myocardial infarction were followed for a median of 3.96 years. Of these, 16.9% received early combination therapy with ezetimibe (≤ 12 weeks after discharge), 18.1% received late combination therapy (13 weeks–16 months), and 65.0% remained on statin monotherapy. Regarding LDL-C goal attainment, the proportion achieving the ESC/EAS target (< 1.4 mmol/L [55 mg/dL]) at one year was approximately 55% among those receiving early combination therapy, 43% with late combination, and 34% with statin monotherapy. During follow-up, 2,570 MACE occurred. The 1-year MACE incidence was 1.79 per 100 patient-years in the early-combination group, compared with 2.58 in the late-combination group and 4.03 in the statin monotherapy group. The corresponding 3-year hazard ratios were 1.14 (95% CI, 0.95–1.41) for late combination and 1.29 (95% CI, 1.12–1.55) for statin monotherapy versus early combination, with lower cardiovascular mortality in the early-treatment group (HR, 1.83 [95% CI, 1.35–2.69] vs. monotherapy). Model-based extrapolation suggested that, if all post-MI patients in the registry had received early statin + ezetimibe, approximately 477 MACE could have been prevented over three years, equivalent to 13–14 fewer events per 1,000 patients treated early. The corresponding number needed to treat (NNT) was 53 (95% CI, 32–125) compared to statin monotherapy and 143 (95% CI, 44–167) compared to late combination therapy.

Following such an approach, some patients with low untreated LDL-C levels might reach very low on-treatment levels under a combination default. The American Guideline explicitly advise against de-escalation at very low LDL-C [11]. This position is supported by robust evidence. In a meta-analysis led by Sabatine, which pooled data from the Cholesterol Treatment Trialists’ Collaboration (26 statin trials, > 170,000 patients) together with three major nonstatin LDL-C lowering trials (IMPROVE-IT, FOURIER, ODYSSEY OUTCOMES; in total > 50,000 patients), the benefit of LDL-C lowering was examined in populations starting at median baseline LDL-C as low as 1.6–1.8 mmol/L (63–70 mg/dL) [30]. Across statin and nonstatin trials, each 1 mmol/L (38.7 mg/dL) LDL-C reduction translated into a ~ 20% relative reduction in major vascular events, with no increase in adverse outcomes, including new-onset diabetes, hepatic enzyme elevations, hemorrhagic stroke, or cancer. Notably, clinical benefit was observed down to achieved median LDL-C levels of ~ 0.5 mmol/L (21 mg/dL) [30].

Thus, available evidence suggests that further lowering of LDL-C beyond current targets continues to reduce cardiovascular risk without offsetting harms. The small increase in new-onset diabetes risk observed with statins appears to be drug class-specific and is outweighed by the reduction in ASCVD events [31]. Moreover, the biological concern of complete cholesterol depletion is theoretical: hepatocytes retain the ability to synthesise cholesterol endogenously, and pharmacological inhibitors of cholesterol synthesis (statins, bempedoic acid) do not completely block this process [32]. Likewise, no evidence links very low LDL-C concentrations to cognitive decline or dementia; in the EBBINGHAUS substudy of FOURIER and subsequent long-term analyses, no neurocognitive or Alzheimer-related adverse effects were observed even at LDL-C levels < 0.5 mmol/L [33, 34].

Taken together, these data support the notion that a “combination for all” approach after ACS may offer a pragmatic and effective strategy to close treatment gaps, with very low LDL-C levels not representing overtreatment but rather an extension of clinical benefit.

## Conclusions

International guidelines endorse lower LDL-C targets and suggest the earlier use of combination therapy; nevertheless, a sequential approach, starting with statins and later stepwise escalation of lipid-lowering therapy, persists in many healthcare systems. This paradigm, while consistent with traditional treatment algorithms, partially contributes to the persistent undertreatment observed in the majority of patients with ASCVD or following ACS, as large proportions fail to achieve guideline-recommended LDL-C levels in real-world practice.

Findings from randomised controlled trials, trial emulations, and observational registries collectively support the notion that a default high-intensity statin plus ezetimibe for ACS patients at discharge might be a pragmatic and implementable step that could reduce delays, increase early goal attainment, and ultimately prevent events. At the same time, while a “combination for all” approach offers a pragmatic and straightforward solution to undertreatment, it runs counter to the broader trajectory of personalised medicine, which seeks to tailor therapy intensity to an individual’s baseline LDL-C, risk profile, and treatment response. Balancing the advantages of simplification with the principles of precision care remains an open challenge for future guideline development and implementation research.

## Key References

- Brandts J, et al. International patterns in lipid management and implications for patients with coronary heart disease: results from the INTERASPIRE study. Eur J Prev Cardiol. 2025; zwaf388.
  - ○ This is the latest multinational survey of patients with established coronary heart disease documents global variations in lipid management and reveals that fewer than one in five achieve ESC/EAS LDL-C targets, highlighting the persistent international treatment gap that motivates the consideration of earlier combination therapy.
- Leosdottir M, Schubert J, Brandts J et al. Early Ezetimibe Initiation After Myocardial Infarction Protects Against Later Cardiovascular Outcomes in the SWEDEHEART Registry.J Am Coll Cardiol. 2025.
  - ○ This nationwide registry analysis shows that early initiation of ezetimibe after myocardial infarction is associated with higher LDL-C target attainment and fewer recurrent cardiovascular events compared with delayed or no combination therapy.
- Haertel F, Makhmudova U, Geiling J-A et al. Less intensive lipid-lowering therapy after ST-elevation myocardial infarction is associated with cardiovascular events: 2-year follow-up of “Jena auf Ziel.” Clin Res Cardiol. 2025.
  - ○ This prospective cohort study demonstrates that early combination therapy achieves near-complete LDL-C target attainment at one year, whereas subsequent de-intensification in community care leads to higher LDL-C levels and increased cardiovascular event rates.

## Data Availability

No datasets were generated or analysed during the current study.

## References

[1] Ulvenstam A, Graipe A, Irewall A-L, Söderström L, Mooe T. Incidence and predictors of cardiovascular outcomes after acute coronary syndrome in a population-based cohort study. Sci Rep. 2023;13(1):3447. 36859606 10.1038/s41598-023-30597-w PMC9977928

[2] Li S, Peng Y, Wang X, Qian Y, Xiang P, Wade SW, Guo H, Lopez JAG, Herzog CA, Handelsman Y. Cardiovascular events and death after myocardial infarction or ischemic stroke in an older medicare population. Clin Cardiol. 2019;42(3):391–9. 30697776 10.1002/clc.23160 PMC6712383

[3] Jernberg T, Hasvold P, Henriksson M, Hjelm H, Thuresson M, Janzon M. Cardiovascular risk in post-myocardial infarction patients: nationwide real world data demonstrate the importance of a long-term perspective. Eur Heart J. 2015;36(19):1163–70. 25586123 10.1093/eurheartj/ehu505

[4] Trialists CT. The effects of Lowering LDL cholesterol with Statin therapy in people at low risk of vascular disease: meta-analysis of individual data from 27 randomised trials. Lancet. 2012;380(9841):581–90. 22607822 10.1016/S0140-6736(12)60367-5 PMC3437972

[5] Koskinas KC, Siontis GCM, Piccolo R, Mavridis D, Räber L, Mach F, Windecker S. Effect of Statins and non-statin LDL-lowering medications on cardiovascular outcomes in secondary prevention: a meta-analysis of randomized trials. Eur Heart J. 2017;39(14):1172–80. 10.1093/eurheartj/ehx566 29069377

[6] Efficacy and safety of LDL-lowering therapy among men and women: meta-analysis of individual data from 174 000 participants in 27 randomised trials. The Lancet 2015, 385(9976):1397–1405. 10.1016/S0140-6736(14)61368-4 25579834

[7] Mach F, Koskinas KC, Roeters van Lennep JE, Tokgözoğlu L, Badimon L, Baigent C, Benn M, Binder CJ, Catapano AL, De Backer GG et al. 2025 focused update of the 2019 ESC/EAS guidelines for the management of dyslipidaemias: developed by the task force for the management of dyslipidaemias of the European society of cardiology (ESC) and the European atherosclerosis society (EAS). Eur Heart J 2025.

[8] Mach F, Baigent C, Catapano AL, Koskinas KC, Casula M, Badimon L, Chapman MJ, De Backer GG, Delgado V, Ference BA, et al. 2019 ESC/EAS guidelines for the management of dyslipidaemias: lipid modification to reduce cardiovascular risk: the task force for the management of dyslipidaemias of the European society of cardiology (ESC) and European atherosclerosis society (EAS). Eur Heart J. 2019;41(1):111–88. 10.1093/eurheartj/ehz455 31504418

[9] Visseren FLJ, Mach F, Smulders YM, Carballo D, Koskinas KC, Bäck M, Benetos A, Biffi A, Boavida J-M, Capodanno D, et al. 2021 ESC guidelines on cardiovascular disease prevention in clinical practice: developed by the task force for cardiovascular disease prevention in clinical practice with representatives of the European society of cardiology and 12 medical societies with the special contribution of the European association of preventive cardiology (EAPC). Eur Heart J. 2021;42(34):3227–337. 34458905 10.1093/eurheartj/ehab484

[10] Byrne RA, Rossello X, Coughlan JJ, Barbato E, Berry C, Chieffo A, Claeys MJ, Dan G-A, Dweck MR, Galbraith M, et al. 2023 ESC guidelines for the management of acute coronary syndromes: developed by the task force on the management of acute coronary syndromes of the European society of cardiology (ESC). Eur Heart J. 2023;44(38):3720–826. 37622654 10.1093/eurheartj/ehad191

[11] Rao SV, O’Donoghue ML, Ruel M, Rab T, Tamis-Holland JE, Alexander JH, Baber U, Baker H, Cohen MG, Cruz-Ruiz M. 2025 ACC/AHA/ACEP/NAEMSP/SCAI guideline for the management of patients with acute coronary syndromes: a report of the American college of Cardiology/American heart association joint committee on clinical practice guidelines. J Am Coll Cardiol. 2025;85(22):2135–237. 40013746 10.1016/j.jacc.2024.11.009

[12] Kim B-K, Hong S-J, Lee Y-J, Hong SJ, Yun KH, Hong B-K, Heo JH, Rha S-W, Cho Y-H, Lee S-J, et al. Long-term efficacy and safety of moderate-intensity Statin with Ezetimibe combination therapy versus high-intensity Statin monotherapy in patients with atherosclerotic cardiovascular disease (RACING): a randomised, open-label, non-inferiority trial. Lancet. 2022;400(10349):380–90. 35863366 10.1016/S0140-6736(22)00916-3

[13] Hong S-J, Lee Y-J, Lee S-J, Hong B-K, Kang WC, Lee J-Y, et al. Treat-to-target or high-intensity statin in patients with coronary artery disease: a randomized clinical trial. JAMA. 2023;329(13):1078–87. 36877807 10.1001/jama.2023.2487 PMC9989958

[14] Schwartz GG, Steg PG, Szarek M, Bhatt DL, Bittner VA, Diaz R, Edelberg JM, Goodman SG, Hanotin C, Harrington RA, et al. Alirocumab and cardiovascular outcomes after acute coronary syndrome. N Engl J Med. 2018;379(22):2097–107. 30403574 10.1056/NEJMoa1801174

[15] Sabatine MS, Giugliano RP, Keech AC, Honarpour N, Wiviott SD, Murphy SA, Kuder JF, Wang H, Liu T, Wasserman SM, et al. Evolocumab and clinical outcomes in patients with cardiovascular disease. N Engl J Med. 2017;376(18):1713–22. 28304224 10.1056/NEJMoa1615664

[16] Nissen SE, Lincoff AM, Brennan D, Ray KK, Mason D, Kastelein JJ, Thompson PD, Libby P, Cho L, Plutzky J. Bempedoic acid and cardiovascular outcomes in Statin-Intolerant patients. N Engl J Med 2023.

[17] Räber L, Ueki Y, Otsuka T, Losdat S, Häner JD, Lonborg J, Fahrni G, Iglesias JF, van Geuns R-J, Ondracek AS, et al. Effect of Alirocumab added to High-Intensity Statin therapy on coronary atherosclerosis in patients with acute myocardial infarction: the PACMAN-AMI randomized clinical trial. JAMA. 2022;327(18):1771–81. 35368058 10.1001/jama.2022.5218 PMC8978048

[18] Nicholls SJ, Kataoka Y, Nissen SE, Prati F, Windecker S, Puri R, Hucko T, Aradi D, Herrman J-PR, Hermanides RS, et al. Effect of Evolocumab on coronary plaque phenotype and burden in Statin-Treated patients following myocardial infarction. JACC: Cardiovasc Imaging. 2022;15(7):1308–21. 35431172 10.1016/j.jcmg.2022.03.002

[19] Anderson KM, Wilson PW, Garrison RJ, Castelli WP. Longitudinal and secular trends in lipoprotein cholesterol measurements in a general population sample the Framingham offspring study. Atherosclerosis. 1987;68(1–2):59–66. 3500729 10.1016/0021-9150(87)90094-3

[20] Gerique JAG, Gonzalez IF, Herrera MAR, Pablos DL, Ballesteros BM, Sardina RG, de la Cámara AG. Improvement of serum lipids concentration in a general population historical cohort. Why? Clínica E Investigación En Arteriosclerosis. 2017;29(6):239–47. 29037827 10.1016/j.arteri.2017.07.001

[21] Ray KK, Molemans B, Schoonen WM, Giovas P, Bray S, Kiru G, Murphy J, Banach M, De Servi S, Gaita D, et al. EU-Wide Cross-Sectional observational study of Lipid-Modifying therapy use in secondary and primary care: the DA VINCI study. Eur J Prev Cardiol. 2020;28(11):1279–89. 10.1093/eurjpc/zwaa047 33580789

[22] Ray KK, Haq I, Bilitou A, Manu MC, Burden A, Aguiar C, et al. Treatment gaps in the implementation of LDL cholesterol control among high-and very high-risk patients in Europe between 2020 and 2021: the multinational observational SANTORINI study *The Lancet Regional Health–Europe*. 2023. 10.1016/j.lanepe.2023.100624. 37090089 10.1016/j.lanepe.2023.100624 PMC10119631

[23] Ray KK, Aguiar C, Arca M, Connolly DL, Eriksson M, Ferrières J, Laufs U, Mostaza JM, Nanchen D, Bardet A. Use of combination therapy is associated with improved LDL cholesterol management: 1-year follow-up results from the European observational SANTORINI study. Eur J Prev Cardiol. 2024;31(15):1792–803. 38861400 10.1093/eurjpc/zwae199

[24] Toplak H, Bilitou A, Alber H, Auer J, Clodi M, Ebenbichler C, Fließer-Görzer E, Gelsinger C, Hanusch U, Ludvik B, et al. Simulation of bempedoic acid and Ezetimibe in the lipid-lowering treatment pathway in Austria using the contemporary SANTORINI cohort of high and very high risk patients. Wiener Klinische Wochenschrift. 2023;135(13):364–74. 37286910 10.1007/s00508-023-02221-4 PMC10338584

[25] Brandts J, Bray S, Villa G, Catapano AL, Poulter NR, Vallejo-Vaz AJ, et al. Optimal implementation of the 2019 ESC/EAS dyslipidaemia guidelines in patients with and without atherosclerotic cardiovascular disease across Europe: A simulation based on the DA VINCI study.The Lancet Regional Health–Europe. 2023. 10.1016/j.lanepe.2023.100665. 37547279 10.1016/j.lanepe.2023.100665 PMC10398584

[26] Brandts J, Barkas F, De Bacquer D, Jennings C, De Backer GG, Kotseva K, Ryden L, Lip GY, Erlund I, Ganly S. International patterns in lipid management and implications for patients with coronary heart disease: results from the INTERASPIRE study. Eur J Prev Cardiol 2025:zwaf388. 10.1093/eurjpc/zwaf388 40796307

[27] Makhmudova U, Samadifar B, Maloku A, Haxhikadrija P, Geiling J-A, Römer R, Lauer B, Möbius-Winkler S, Otto S, Schulze PC, et al. Intensive lipid-lowering therapy for early achievement of guideline-recommended LDL-cholesterol levels in patients with ST-elevation myocardial infarction (Jena auf Ziel). Clin Res Cardiol. 2023;112(9):1212–9. 36602598 10.1007/s00392-022-02147-3 PMC10449699

[28] Haertel F, Makhmudova U, Geiling J-A, Lauer B, Möbius-Winkler S, Otto S, Schulze PC, Weingärtner O. Less intensive lipid-lowering therapy after ST-elevation myocardial infarction is associated with cardiovascular events: 2-year follow-up of Jena auf Ziel. Clin Res Cardiol 2025. 10.1007/s00392-025-02736-y PMC12823638 40864246

[29] Margret Leosdottir M, PHD,a,b Jessica Schubert, MD,c Julia Brandts, MD,d,e Stefan Gustafsson, PHD,f, Thomas Cars P, f Johan Sundström, MD, PHD,c Tomas Jernberg, MD, PHD,g Kausik, Ray K. MBCHB, MD, FMEDSCI,d,h, Emil Hagström M, PHDc,: Early Ezetimibe Initiation After Myocardial Infarction Protects Against Later Cardiovascular Outcomes in the SWEDEHEART Registry. J Am Coll Cardiol 2025, in press. 10.1016/j.jacc.2025.02.007 40240093

[30] Sabatine MS, Wiviott SD, Im K, Murphy SA, Giugliano RP. Efficacy and safety of further lowering of low-Density lipoprotein cholesterol in patients starting with very low levels: a meta-analysis. JAMA Cardiol. 2018;3(9):823–8. 30073316 10.1001/jamacardio.2018.2258 PMC6233651

[31] Brandts J, Müller-Wieland D. Debate: lipid-lowering therapies and diabetes development. Curr Atheroscler Rep. 2024;27(1):24. 10.1007/s11883-024-01270-y PMC11711849 39775321

[32] Goldstein JL, Brown MS. A century of cholesterol and coronaries: from plaques to genes to statins. Cell. 2015;161(1):161–72. 25815993 10.1016/j.cell.2015.01.036 PMC4525717

[33] Giugliano RP, Mach F, Zavitz K, Kurtz C, Im K, Kanevsky E, Schneider J, Wang H, Keech A, Pedersen TR, et al. Cognitive function in a randomized trial of Evolocumab. N Engl J Med. 2017;377(7):633–43. 28813214 10.1056/NEJMoa1701131

[34] Zimerman A, O’Donoghue ML, Ran X, Im K, Ott BR, Mach F, Zavitz K, Kurtz CE, Monsalvo ML, Wang B. Long-term cognitive safety of achieving very low LDL cholesterol with Evolocumab. NEJM Evid. 2025;4(1):EVIDoa2400112. 39718423 10.1056/EVIDoa2400112

